# Hospital and Health News

**Published:** 1924-07

**Authors:** 


### HOSPITAL AND HEALTH NEWS.

### Hospital Sunday.

Hospital Sunday in London was observed on June 22. This is the fifty-second year of the Fund, which has distributed in all £2,842,000 since its foundation.

### A Presentation.

Mr. H. H. Collier, who till his recent resignation, has been honorary secretary to Acton Hospital for six years, has been presented with a silver inkstand and a hall barometer.

### Bust of John Hunter.

It is proposed to place the bronze bust of John Hunter, by Gilbert, belonging to St. George's Hospital, on a pier in the railings fronting the hospital extension in Knightsbridge.

### Twenty-four Years as Chairman.

With his recent re-election Mr. P. B. Burgoyne enters on his twenty-fourth consecutive year of office as chairman of the Royal National Hospital for Consumption, Ventnor.

### A Woman on the B.M.A. Council.

Dr. Christine Murrell has been elected to the Council of the British Medical Association. This is the first time a woman has sat on the Council.

### A New Address.

The address of the General Nursing Council for Scotland is now 18 Melville Street, Edinburgh, not 13 Melville Street, as hitherto.

### "The World's Health."

Dr. Claude Lillingston, who, it will be remembered, was one of the Consolation Prize Winners in our recent Health Week Competition, has been appointed editor of "The World's Health," the monthly journal published in Paris by the League of Red Cross Societies.

### Chairman's Gift to a Hospital.

Mr. Leonard Ropner, Chairman of the Stockton and Thornaby Hospital, has given £10,000 toward the cost of the extensions. The scheme includes two medical wards, a children's ward, an out-patient department and a nurses' home, and apart from his gift £18,000 is in hand towards it.

### Two New Secretaries.

Capt. A. Wright, D.S.O., is the new secretary to the East Sussex Hospital, Hastings. Previously secretary to All Saints Hospital, London, Capt. Wright was on the secretarial staff of St. Thomas's. Mr. E. D. West has succeeded Mr. W. H. Head as Financial Secretary to the Bognor War Memorial Hospital.

### Lectures at the Infants' Hospital.

A course of lectures for Health Visitors, Nurses and others interested in Maternity and Child Welfare will be given at the Infants' Hospital, Vincent Square, Westminster, S.W., at 6 p.m., on Wednesdays, from July 2 to September 17. Tickets for the course are 5s., and are obtainable from the Secretary of the Hospital.

### Porter of the London Hospital Retires.

Mr. John Burridge, the porter of the London Hospital, is retiring at the age of seventy. He has been in the service of the hospital for fifty years. When he joined the staff he began to work in the basement, but his familiar figure has been seen in the hall for forty years. His most vivid memories perhaps concern the war, when one of the air-raids brought over two hundred victims to the hospital.

### River Trips for Civilian Patients.

The plan of giving river trips to Kew or Southend to soldiers and sailors in London hospitals is to be extended to convalescents in civil hospitals and infirmaries if enough support is forthcoming. The Rev. John Stanley, who has organised the work since 1914, invites co-operation, and all who wish to help should notify him at 15 Rue du Duc, Avenue Tervueren, Brussels. He will then form a committee to carry the scheme through. The cost of a trip works out at 2s. 6d. a head; £5 will provide for fifty, and £20 pays for a whole trip, which can be taken in the name of the donor who may accompany the party.

### The Chemicalisation of Flour.

The Minister of Health has extended the terms of reference of the Departmental Committee on the use of preservatives and colouring matters in food so as to include the question whether, and to what extent, the practice of treating flour with chemical substances is objectionable on grounds of health, and whether it is desirable in the interest of the public health that the practice should be prohibited or restricted, and in the latter case what restrictions should be imposed. The Secretary of the Committee is Mr. A. M. Legge, of the Ministry of Health, Whitehall, S.W., to whom all communications should be addressed.

### Awards to be Won.

The National Baby Week Council will allot a series of awards offered by the *Daily News* to places which show the greatest reduction of the death rate of these children in 1924 as compared with the average in the same places in the previous three years. The awards are: One of £25 and one of £10 to towns of 50,000 population and upwards; one of £25 and one of £10 to districts between 20,000 and 50,000; and one of £25 and one of £10 to districts between 5,000 and 20,000. Details may be obtained by sending a stamped addressed envelope to the National Baby Week Council, 117, Piccadilly, W. 1.

### To Remove a Stigma.

The Metropolitan Asylums Board has decided to ask the Minister of Health to issue an order making aged poor persons, suffering from senile decay, chargeable to Metropolitan Boards of Guardians, a class for which accommodation may be provided by the Asylums Board under the Metropolitan Poor Act of 1867. Such people, it is argued, though not lunatics, are certified as such in their old age, and the idea is to remove the stigma of lunacy from them.

### Committee on Hospital Services in Scotland.

The Scottish Board of Health have appointed Lord Mackenzie, Sir Norman Walker, Mr. D. J. Mackintosh, Mr. T. J. Addly, Mr. John Birnie, Dr. R. C. Buist, Dr. A. K. Chalmers, Mr. John S. Doeg, Mr. Russell Paton, Mr. James Robertson, Mrs. Elizabeth Shirley, and Mr. Joseph Waugh, to be a Committee to inquire into and report upon the extent and nature of the inadequacy of the present hospital and ancillary services in Scotland, and to make recommendations for the development and maintenance of those services to meet the needs of the community. Lord Mackenzie is Chairman and Mr. Niven McNicoll Secretary.

### Bequests for Hospitals.

Six London hospitals receive sums of £6,000 apiece under the will of Mr. Sydney Larnach, of East Grinstead, who left over a million and a-quarter. Almost simultaneously the will is announced of the late Sir John Bell, who was Lord Mayor of London 1907-8, and left half a million. He bequeathed one-half of the ultimate residue of his estate, "provided the control and management of hospitals should not be taken over by H.M.'s Government or the expenses of upkeep of hospitals thrown upon either the rates or the taxes," for division equally among the King's Fund, the Sunday Fund and two homes for incurables. The extension of Bolingbroke Hospital, Wandsworth Common, at a cost of £50,000, has been made possible by the generosity of the late Mr, W. Shepherd's trustees, who have provided £35,000.

### A Conference on Infant Welfare.

From July 1 to 4 at the Caxton Hall, Westminster, the National League for Health Maternity and Child Welfare will hold its third English-speaking conference. A presidential address will be given on the first day by the Minister of Health, while on subsequent days the chair will be taken by Mr. Neville Chamberlain; Surgeon S. L. Christian, representative of the Government for the U.S.A.; the Hon. Mrs. Eustace Hills; Dr. E. W. Morris, representative for the Commonwealth of Australia; the Viscountess Rhondda. A number of tours by motor have been arranged to various hospitals and welfare centres, a mothercraft exhibition will be open daily at 117 Piccadilly, a bookstall for the sale and study of recent publications connected with the Child Welfare movement will be open at Caxton Hall, and as far as possible delegates will be helped to secure accommodation. Further particulars and application forms may be obtained from the Secretary, Carnegie House, 117 Piccadilly.

### The City of London Coroner's Report.

In his report for 1923 Dr. Waldo writes that in only 56 cases of public inquests coming before him was a jury dispensed with in accordance with the still existing special war legislation. "I am of opinion that in the Coroner's Court, the best tribunal for the elicitation of truth is made up of a jury directed by a Coroner, and that the only cases in which a Coroner's discretionary power to sit without a jury can properly be exercised in the public interest, are those of natural as opposed to violent deaths. As matters are at present the former uniformity of practice, which marked the Coroner's Court in the calling of a jury by the Coroner at every inquest, has disappeared, and with it the general view of the body, save by the Coroner."

### Women's Holiday Fund.

The Bishop of London and others are appealing on behalf of the Women's Holiday Fund. A holiday to many of the women living in the poorer districts of London is an utter impossibility, for it is too much of a luxury to be paid for out of the weekly money which has to be so carefully laid out to meet even the necessaries of every day life. Among the 200 applications already received this year, 20 applicants have never had a holiday in their lives. The approximate cost of a fortnight's holiday is £3 or £3 10s. for a mother with a baby towards which the women contribute on an average less than one-third. Donations should be sent to the Secretary Women's Holiday Fund, 76, Denison House, 296, Vauxhall Bridge-road, S.W. 1.

### Royal Sanitary Institute Congress.

The Thirty-fifth Congress of the Royal Sanitary Institute will be held at Liverpool from July 14 to 19. Already 500 delegates from 275 authorities representing Foreign and Colonial Governments, Government Departments, County Councils, etc., have been appointed. The Marquess of Salisbury, as President of the Congress, will deliver his address at the inaugural meeting in the St. George's Hall on July 14 at 8.30 p.m. The Congress is divided into sections and conferences, and among the subjects to be discussed are cancer, diphtheria, ante-natal clinics, child welfare, dental and school medical work; clean milk, handling of food, meat inspection and training of inspectors; housing, regional surveys, arterial roads, town planning and water supply; industrial welfare, lighting of factories, ship sanitation, work of health visitors, school nurses and midwives.

### Women Doctors' Jubilee.

The London (Royal Free Hospital) School of Medicine for Women, part of the University of London, was opened in October, 1874, and the jubilee of this movement is to be celebrated in October by a thanksgiving service in St. Paul's Cathedral and by a festival dinner in the Guildhall. As a permanent memorial of this event it is hoped that fifty representative women will each give or collect £1,000 to endow three chairs at the Medical School in Hunter Street, Bloomsbury. These chairs will bear the names of the founders of the school, the pioneers of this great step in the progress of women's work. These ladies were Dr. Elizabeth Blackwell, English born but who obtained her M.D. degree in America; Miss Sophia Jex-Blake, who, in 1869, led the endeavour of women to enter Edinburgh University as medical students; and Dr. Elizabeth Garrett Anderson, who, in 1865, was the first woman to secure a licence to practise medicine in this country.

### Tuberculosis Conferences.

On Thursday, July 3, and Friday, July 4, the 10th Annual Conference of the National Association for the Prevention of Tuberculosis will be held. On the Thursday there will be demonstrations at Burrow Hill Training Colony, Frimley, Surrey, after which delegates will be invited to ask questions. Delegates are asked to assemble at 20, Hanover Square, not later than 9.15 a.m. to be conveyed, free of charge, to Frimley by char-à-bancs. Lunch and tea will be served at the colony and chars-à-bancs will arrive back in London at 7 p.m. On the Friday there will be a conference at Robert Barnes Hall, 1, Wimpole Street, to be opened by the Minister of Health, when a discussion on "The Part Played by Training Colonies in the Treatment of Tuberculosis" will be opened by Dr. F. N. K. Menzies. In the afternoon, at 2.30, the Annual Meeting of the Association will be held. This will be followed by a Conference of Care Committees.

